# Measuring CO_2_ and CH_4_ with a portable gas analyzer: Closed-loop operation, optimization and assessment

**DOI:** 10.1371/journal.pone.0193973

**Published:** 2018-04-04

**Authors:** Jeremy Wilkinson, Christoph Bors, Florian Burgis, Andreas Lorke, Pascal Bodmer

**Affiliations:** Institute for Environmental Sciences, University of Koblenz-Landau, Landau, Germany; Tallinn University of Technology, ESTONIA

## Abstract

The use of cavity ring-down spectrometer (CRDS) based portable greenhouse gas analyzers (PGAs) in closed-loop configuration to measure small sample volumes (< 1 l) for CH_4_ and CO_2_ concentrations is increasing and offers certain advantages over conventional measurement methods in terms of speed as well as the ability to measure directly in field locations. This first systematic assessment of the uncertainties, problems and issues associated with achieving reliable and repeatable measurement with this technique presents the adaptation, measurement range, calibration and maintenance, accuracy and issues of efficient operation, for one example instrument. Regular open-loop calibration, a precise loop volume estimate, leak free system, and a high standard of injection practices are necessary for accurate results. For 100 μl injections, measured values ranging from 4.5 to 9 x10^4^ ppm (CH_4_), and 1000 ppm to 1 x10^6^ ppm (CO_2_) are possible with uncertainties ±5.9% and ±3.0%, respectively, beyond 100 ppm CH_4_ correction may be necessary. Uncertainty arising from variations water vapour content and atmospheric pressure are small (0.24% and -0.9% to +0.5%, respectively). With good practice, individual operator repeatability of 1.9% (CH_4_) and 2.48% (CO_2_) can be achieved. Between operator injection error was around 3% for both gases for four operators. Slow syringe plunger operation (> 1s) is recommended; generally delivered more (ca. 3–4%) sample into the closed instrument loop than did rapid operation. Automated value retrieval is recommended; we achieved a 3 to 5-fold time reduction for each injection cycle (ca. <2 min), and operator reading, recording, and digitization errors are eliminated.

## 1. Introduction

The accelerating increase of atmospheric concentrations of carbon dioxide (CO_2_) and methane (CH_4_) are the major drivers for current and projected climate change [1]. Global budgets of both these greenhouse gases (GHGs) are relatively well constrained, but the distribution and dynamics of sources and sinks, their vulnerability to anthropogenic activity [2] and potential feedback responses to changing climatic conditions, are not—particularly at regional and short-time scales [3,4]. Consequently, there is an urgent need for more extensive observations to improve process-based understanding of both natural and anthropogenic sources and sinks of both gases in a wide range of environments and situations.

Until recently, gas mixing ratio or partial pressure (from here on abbreviated as PP) in part per million [ppm], parts per billion [ppb], or parts per thousand [ppt]) of CO_2_ and CH_4_ were generally analyzed by gas chromatography (GC) with flame-ionization detection (FID) for CH_4_, or electron capture detector (ECD) for CO_2_ [5,6]. Modified GC configurations enable combined detector measurement of both GHGs with high accuracy and excellent peak area repeatability [7]. GC measurement provides e.g., a detection limit for the headspace method [8] of up to 2 ppb for CH_4_ also for small gas sample volumes of 100 μl [9], but are generally limited to laboratory use, often requiring prolonged sample storage and transport times and potential sample deterioration prior to analysis, such as sample dilution due to leakage, and biochemical degradation or transformation. Portable GC instruments are available, however, they still need to be calibrated before each measurement, which requires calibration gases and the instruments are generally sensitive to, e.g., leakage [10]. Reactivity must be inhibited by using chemicals, which may alter the pH of water samples, and in turn carbonate equilibrium, or may not completely inhibit biological activity. Thus, delayed analysis prohibits near-real time adaption of sampling strategies and/or sampling frequency. In addition, the use of a carrier gas and other consumables with GC operation generates running costs, which may limit the scope of their use in any study with limited financial resources.

The main alternative measurement technique for gaseous CO_2_ and CH_4_ is laser-based cavity ring-down spectroscopy (CRDS)[6], applied in the form of the portable infrared gas analyser (PGA or (P)IRGA) is capable of providing real time, in-situ measurement of gas PP, and at remote field sites [11–14]. A recent detailed study of GC-FID and CRDS accuracy concluded that CRDS based systems (e.g., PGA) provide direct (open-loop) CO_2_ and CH_4_ measurements of sufficient accuracy, and at higher temporal data coverage, with better linearity and repeatability, without the need for air drying, and can potentially replace GC-FID and extend the global network for GHG observation[6].

PGAs have generally been used to assess natural and anthropogenic sources or sinks of both gases [11,15–17] with eddy-covariance flux measurement [14,18], as well as for headspace gas measurement in closed chamber applications in terrestrial- and aquatic-environments [e.g., 12,13,19,20–22]. High measurement accuracy in combination with versatile and low-power instrument design can be achieved by a variety of spectroscopic techniques, including off-axis cavity-enhanced laser absorption spectrometry such as applied in this study [23,24]. The combination of off-axis laser light (at two frequencies corresponding to absorption by CH_4_, and CO_2_ and water vapour) pulsed through a one way mirror into a cylindrical cavity with a second mirror at the other end, is the key to the success of the technique. The intensity of the light exiting the far-end of the chamber is then focused on and measured by a detector. A partial vacuum is maintained in the chamber to reduce the interference of water vapour. The nature of the enhanced mirror/cavity system means that a very long absorption pathway can be built into a compact and highly portable instrument.

In most applications, PGA have been operated either in open loop for atmospheric gas measurement, or as an integral part of a closed-loop system, such as chamber or aquarium applications [e.g. 12,25,26,27]. Increasingly, PGAs are being used for concentration measurement in small-volume gas samples where the total available sample may only be a few 10s of ml, and only a small sub-sample may be extracted (typically 100 μl)[28], from headspace equilibration of porewater samples [29] and from repetitive sampling of incubation flasks in process-based laboratory tests [28] and from gas bubble development experiments [30]. Since the total effective internal instrument volume may be in the order of 100 ml, the sample must be circulated to obtain a steady concentration, which is achieved by operating in closed-loop configuration (see below for details). The injected sample gas PP is estimated from the observed increase in concentration accounting for dilution by the total closed-loop volume [31]. Depending on the required accuracy, this measurement technique offers an alternative to GC analysis, with the advantages of providing immediate analysis results in field applications with minimal running analysis costs. New developments in IRGAs include hollow fibre-based detection systems [32], with internal volume of around 0.5 ml, and offering detection sensitivity in the pico-gram range, these will no doubt offer fresh possibilities for GHG research.

Comparative uncertainty and accuracy analysis of GC and CRDS has shown very similar results [6,33], however, to our knowledge a systematic assessment of CH_4_ and CO_2_ concentration measurement by PGA in closed-loop has not been performed. Hence, the aims of this article are to describe the adaptation for closed-loop operation and measurement, discuss technical aspects for essential maintenance and calibration, assess closed-loop measurement range, accuracy and precision, and, identify operational efficiencies and optimized data processing. In this way, we present the first technical note supporting PGA users for optimal and satisfactory measurement of small gas volumes with the closed-loop technique. We refer to measurement with a specific portable gas analyzer (UGGA, Los Gatos Research Inc.), however, the test procedures presented can easily be applied to other CRDS-based instruments, and aspects relating to sample injection are relevant to a wide range of instruments where manual injection is undertaken (e.g., portable GC).

## 2. Closed-loop application

### 2.1. Instrument basics

**Fig 1:**
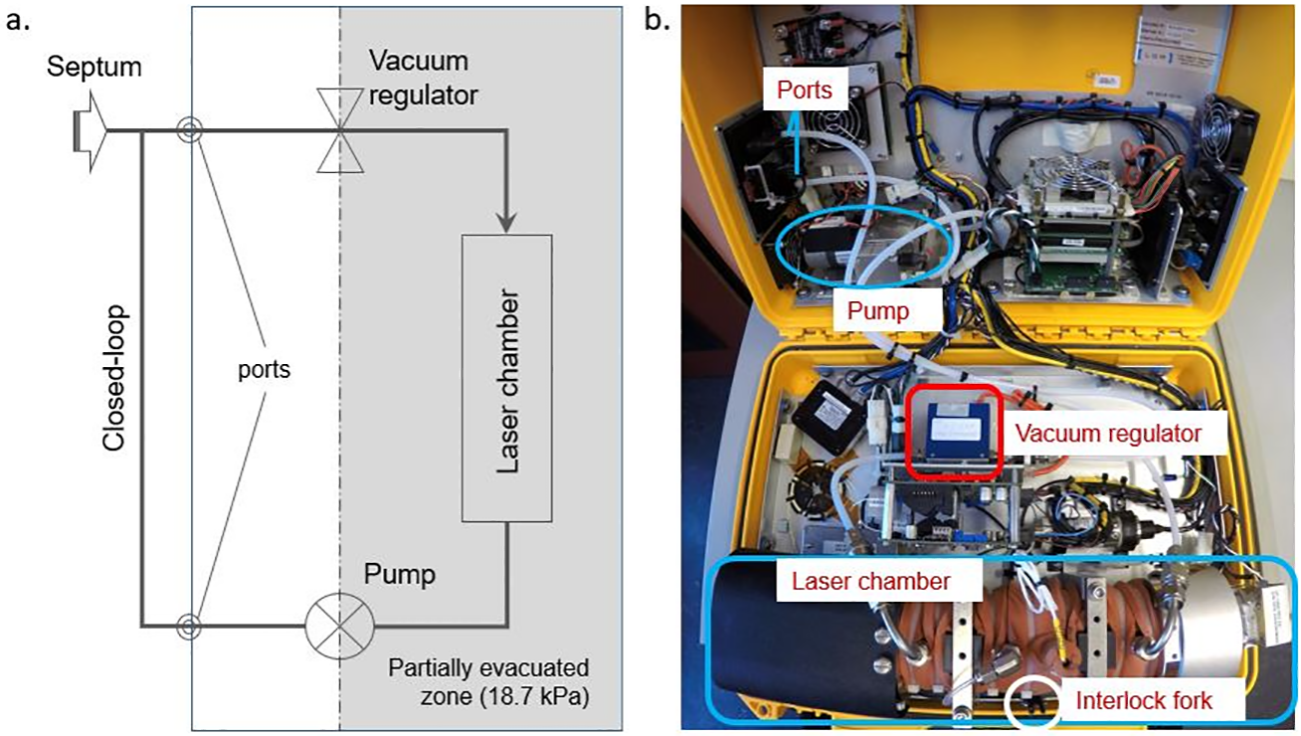
The UGGA system; a. simplified schematic of the UGGA gas loop (shaded area is the partially evacuated loop zone), and b.instrument case interior (note: image distorted due to wide-angle lens).

We present data from three identical PGAs (Ultra-portable Greenhouse Gas Analyzer (UGGA), model 915–0011, Los Gatos Research Inc., Mountain View, Calif., USA) with nominal measurement range 1 to 20000 ppm for CO_2_, 0.01 to 100 ppm for CH_4_, and 500 to 70000 ppm for water vapor, and manufacturer quoted accuracies of ±300 ppb (CO_2_), ±2 ppb (CH_4_) and ±100 ppm (H_2_O). The maximum data recording frequency is 1 Hz. The main components of the instrument internal gas loop are the laser chamber, a circulation pump and a vacuum regulator (Fig 1). The pump has a nominal flow rate of 0.5 l/min. To protect the mirrors the gas loop includes a particle filter. The partial vacuum (18.7 kPa) in the laser chamber is maintained by the flow regulator combined with a one way valve. Instrument real-time output can be displayed by wireless connection to a laptop, tablet, or smart-phone, or directly connection to a monitor screen. Data is stored internally and can be transferred to a portable storage device as required.

### 2.2. Closed-loop adaptation

**Fig 2:**
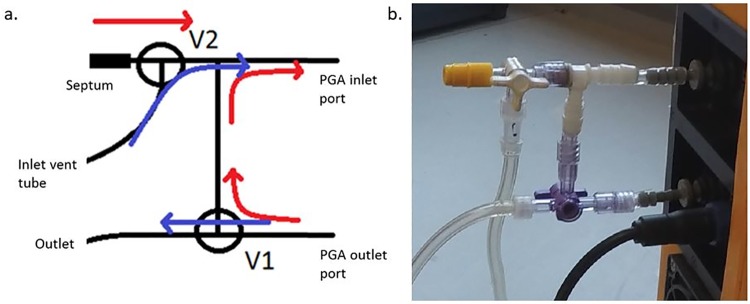
Schematic a. and image b. of the closed gas loop. The valves V1 and V2 are used for switching between venting, i.e., flushing the system with ambient air in (blue arrows) or closed-loop for sample testing (red arrows). The septum is used for sample injection using a syringe.

**Fig 3:**
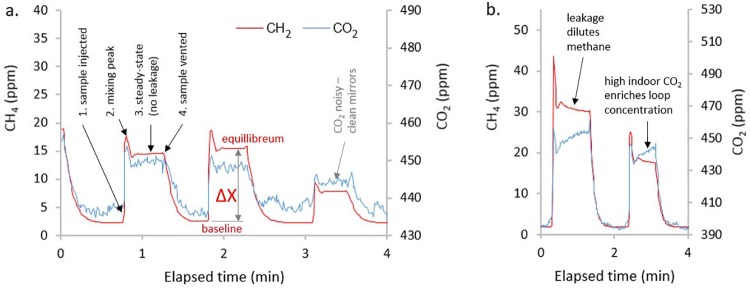
Data excerpts showing a. stages of a closed-loop measurement (without leakage, but noisy CO_2_ response suggesting mirror cleaning needed), and b. a leaking system; the pump draws ambient laboratory air into the partially evacuated circuit diluting methane (red), and increasing CO_2_ (blue) due to indoor air with elevated CO_2_.

Our closed-loop PGA application (Fig 2) consists of an injection cap or septum (Fresenius Kabi AG, 8501502), two 3-way valves (V1 and V2, Fig 2a; Fresenius Kabi AG 8501722), and tee-piece (Rotilabo T-Stück: Roth, E7631) connected with Tygon E3036 tubing which has low permeability for CO_2_ and CH_4_. Valves V1 and V2 are switched between internal circulation during sample injection and measurement (red arrows Fig 2a), and sample venting from the gas loop with ambient air (blue arrows Fig 2a). We vent with outdoor air via a long tube (ca. 3 m) to maintain stable baseline CO_2_ (see Fig 3a) compared to indoor air, where mainly CO_2_ can rise and fall dramatically.

### 2.3. Instrument response and sample value calculation

Upon sample injection (Fig 3a[1]), the instrument responds rapidly with a concentration peak (Fig 3a[2]) as the sample circulates. Complete mixing is achieved after around 12 s (Fig 3a[3]). We recommend leaving the sample circulating for 15–30 s to establish a steady equilibrium value. The time for flushing with ambient air until a stable background reading is established is approximately 45 s (for operational efficiency see subsection 5.1).

The gas concentrations (PP of X_CH4_ and X_CO2_ in ppm) in the injected gas sample (X_*sample*_) can be calculated from the difference between the average equilibrium and baseline values (ΔX, Fig 3a) by:

$$X_{sample}=\Delta X\frac{V_{loop}+V_{sample}}{V_{sample}}$$ (1)

Where, *V*_*loop*_ is the sum of the internal loop volume of the instrument (around 100 mL, see 3.2) and the volume of the external loop connection (approximately 2 mL). The loop volume for each instrument will be slightly different and should be estimated for each instrument as described in 3.2.

### 2.4. Measurement range

**Fig 4:**
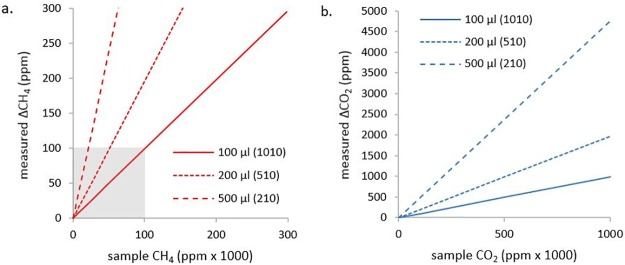
a. and b. measured ΔX (a. CH_4_ and b. CO_2_) concentration (y-axis) vs. sample concentration (x-axis) for three injection volumes 100, 200 and 500 μl, respectively. Legend values in brackets are the dilution factor (V_loop_-V_sample_)/V_sample_. The shaded area in (a.) shows the linear response range for CH_4_.

Closed-loop measurement range is determined by the loop volume (3.2 below) and injection volume since these determine sample dilution (Fig 4). Loop dilution factor is around 900 for a sample volume of 100 μl, this translates into an effective sample value detection range of 4.5 ppm < X_CH4_ < 9 x10^4^ ppm (linear response ≤ 100 ppm, see 4.1 below), 1000 ppm < X_CO2_< 1 x10^6^ ppm, e.g., a 500 μl injection increases the measurement sensitivity by a factor of 5 (Fig 4).

## 3. Calibration and maintenance

### 3.1. Instrument calibration

To ensure continued measurement accuracy and reliability, regular UGGA open-loop testing and recalibration as required is recommended. A reduction in ring-down time (see 3.3) also indicates the need for mirror cleaning, and the disturbance of the system also demands checking and possible recalibration. A one-point calibration is made, thus a single standard gas concentration is required. We test and recalibrate with a standard gas mixture of 80 ppm CH_4_ (within the linear range see 4.1) and 15x10^3^ ppm CO_2_ in nitrogen (part no. 109990011, Messer Schweiz AG company) held in a pre-evacuated gasbag (Tedlar Gas sampling Bag, Supelco, 2L). Calibration requires a constant flow of calibration gas for 2–3 minutes, so at least 2 L of calibration gas. A post-calibration check is made running the same or other standards in open-loop under normal operation from a gasbag.

### 3.2. Loop mixing volume

The instrumental measurement loop volume (V_loop_; ml) is a key parameter for closed-loop measurement, and is determined using standard gases and rearranging Eq (1), such that

$$V_{loop}=\frac{X_{sample}}{\Delta X}V_{sample}{-V}_{sample}$$ (2)

X_*sample*_ is the PP of the injected standard gas (ppm), ΔX is the difference between baseline and equilibrium PP (Fig 4a), and *V*_*sample*_ is the standard gas injection volume (ml). Ideally 100 μl of 10 ppt methane standard is injected into the loop giving ΔX of ca. 10 ppm (equilibrium value of ca. 12 ppm), this is repeated 5–6 times and the mean loop volume computed. We have also calculated the overall long-term mean value of loop volume estimates (see 4.2).

### 3.3. Mirror cleaning and laser ring-down

**Fig 5:**
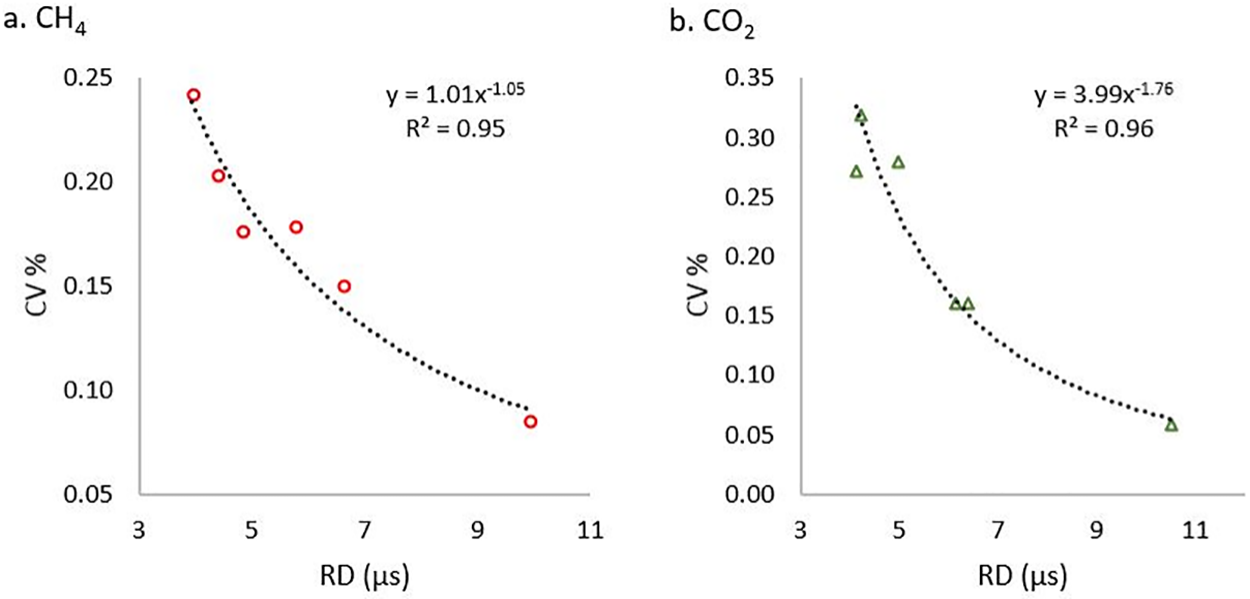
Coefficient of variation (CV) of a. CH_4_ and b. CO_2_ instrument readings in ambient air as a function of ring down time (RD). The data were obtained from different instruments, the dotted lines show least-square power-law fits of the data to RD.

As a data quality indicator, the UGGA records laser cavity ring-down time (RD) at each measurement, this is the characteristic time of the exponential decay of laser energy with repeated reflections within the cavity. Long RD (ca. 9 μs) are required for low data noise (Fig 5). As RD declines with time and use, due to mirror clouding, the increase in measurement noise reduces the ability to detect low sample concentrations. To examine the noise level, we extracted periods of data for ambient air measurement. Signal-to-noise was determined from the residuals of the smoothed data (smoothing as per Wilkinson et al., [28], with smoothing time constant τ = 1.67 min), i.e., raw data minus smoothed values. The coefficient of variation (CV) was determined from the standard deviation of the residual, divided by the mean of the raw data. Measurement with different instruments, showed that the CV was < 0.1% for RD ≥ 9 μs, but increased two to three-fold for RD < 5 μs (Fig 5) (and up to 1.7% for CO_2_ after drying but not cleaning following water ingress into the instrument; RD < 4, see supplement S4 Fig). During data post-processing (see 5 below) the effect of the noise can be reduced by smoothing. For the Los Gatos instruments, a mirror and cell cleaning kit and instructions are available on request [34]. Carefully following this advice have been able to recover RD to the values expected in a new instrument, i.e. RD > 9.

### 3.4. Leakage

Our experience has been that leakage within the closed-loop system generally results in sample dilution or enrichment by ambient air drawn into the instrument (e.g., Fig 3b). The leaks usually originate in the partially evacuated section of the loop (Fig 1a). We have observed leakage in the three instruments we use, and through a process of elimination we were able to identify and seal them. Causes of leakage included loose tubing unions and other joint issues, less obvious were leaks where ambient air from within the instrument case was being drawn into the pump past the two diaphragms. With a closed-loop, leaks increase the total mass of gas in the loop, and consequently, the non-evacuated part of the loop becomes pressurized. This can affect other potential uses of the instrument such as for gas exchange experiments with diffusion tube immersed in a sample water body. Pump leakage can be fixed by greasing the seating of each diaphragm with silicon grease (silicone compound Part No. 10–568, GC Electronics), and Los Gatos inc. recommend a maximum operation time of 1500 h for pump diaphragms; one aged diaphragm set had a reduction in seal lip thickness of 0.4 mm, requiring replacement.

A further cause of leakage found in one instrument resulted from mirror cleaning. The delicate mirror edges can easily suffer edge chipping if not handled with great care during removal and replacement. Such damage may not impact the optical quality of the mirror if outside the limits of the exposed circumference. This leak, although initially difficult to identify, was easily remedied with a small quantity of sealant on the mirror edge during reassembly (note: care must be taken not to smear the optical surfaces).

To facilitate leak detection, exhaled air is blown gently for a few seconds at each joint and component of the gas loop using a narrow tube while watching the instrument response. A leak will be apparent from a spike in CO_2_ response. To do this the interlock switch-fork (Fig 1b) must be removed to operate the instrument with the casing open, and appropriate care should be taken to prevent laser exposure.

### 3.5. Syringe care

Good syringe care and operation is essential for precise closed-loop gas measurement as our data suggest (see 4.4 below). The company Hamilton, for example, offer a “Syringe Care and Use Guide” highlighting necessary actions related to this issue [35]. Syringe plungers should be cleaned and moistened regularly to avoid wear of the inner glass surface due to dust. Partial needle blockage can reduce the sample volume finally injected in to the closed-loop, and sediment residues from sample bottles, and septum rubber fragments were suspected causes of needle blockage in our experience. If a syringe is in good condition, a blockage may cause a partial vacuum inside the syringe resulting and an incomplete sample is drawn. If the syringe is old and worn, ambient air can pass the plunger as it is withdrawn. The opposite effects occur on sample injection into the instrument, resulting in further sample loss, and these two problems can amplify one another. We provide data on measurement variability due to operator and syringe in Section 4.5.

## 4. Measurement uncertainty

In the following section, measurement error ε(%) is used to express the difference between measured (X_meas_) and expected (X_exp_) values (in ppm) as a proportion of X_exp_;

$$\varepsilon (\%)=100\frac{X_{meas}-X_{exp}}{X_{exp}}$$ (3)

The expected value is estimated using mean loop volume (V_loop_) for the given instrument;

$$X_{exp}=X_{stn}\frac{V_{loop}+V_{samp}}{V_{loop}}$$ (4)

Where, X_stn_ is the standard gas PP (ppm) and V_samp_ is the volume of sample injected into the loop.

### 4.1. Methane linear response range

**Fig 6:**
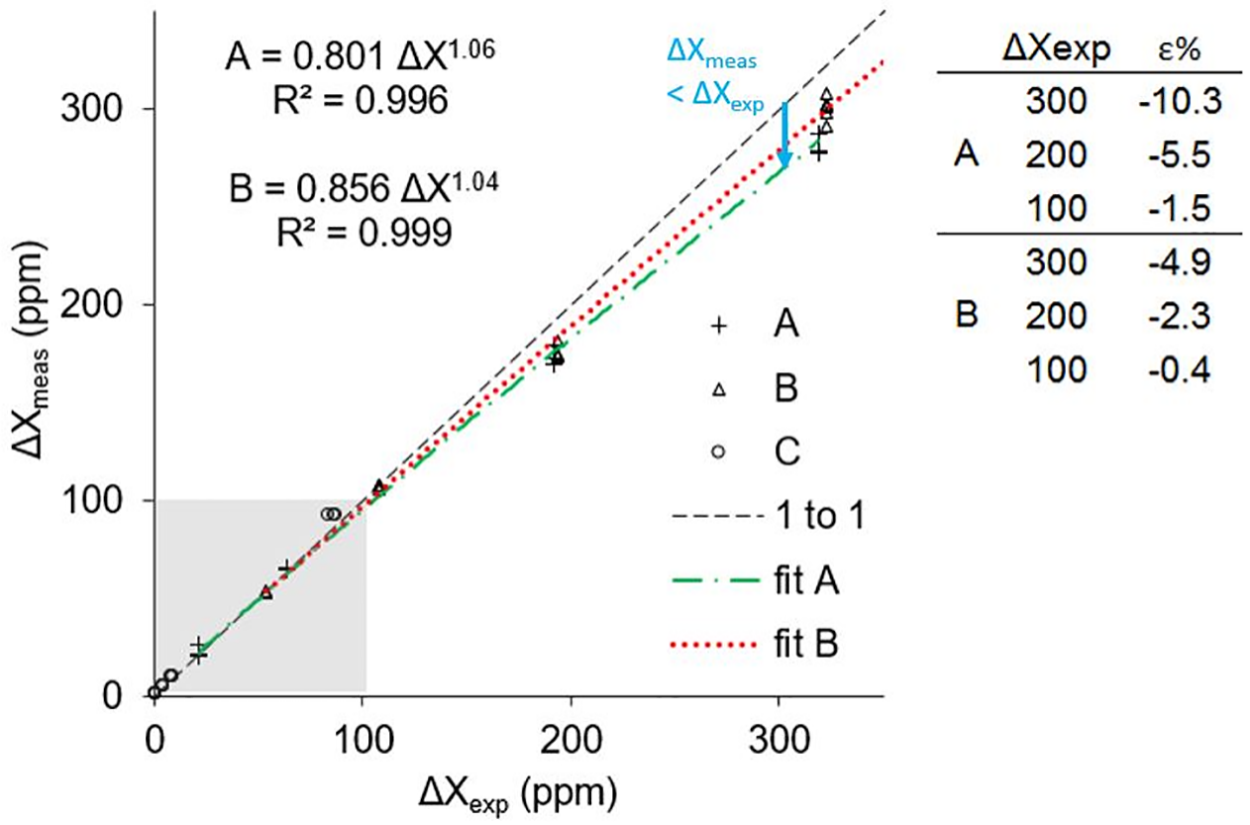
Measured and expected methane ΔX demonstrating instrumental drop-off (indicated by arrow) for X_CH4_ values > 100 ppm (shaded area = linear range). Data for three instruments A, B, and C. Equations for A and B give the correction from measured to expected values. The table inset shows percentage error for A and B.

The nominal measurement range with linear response of our instruments is 100 ppm for methane, beyond which an adjustment may be necessary if a drop-off in response is observed (e.g., Fig 6). We observed drop-off at CH_4_ > 100 ppm for two of our instruments (Fig 6; -10.3% and -4.87% at 300 ppm for A and B respectively, instrument C has not been tested above 100 ppm CH_4_), and an instrument specific correction can be applied (Fig 6). The CO_2_ response of two instruments was tested up to around 700 and 1400 ppm, respectively (using injections of between 20 and 100 μl of standard gases of 63 and 950 ppt) no drop-off in response was evident.

### 4.2. Water vapour

Most gaseous samples will contain some water vapour (for the relative humidity range of 40 to 75%, water vapor content is 1.1 to 1.7%), the UGGA is, however, not routinely calibrated for water vapour. The instrument firmware makes a correction for water vapour (due to dilution) to CO_2_ and CH_4_ PP (X_dry_ = X_wet_ / (1-[H2O]/1e^6^), where [H2O] is the vapour PP in ppm), and the instrument reports both dry and wet mole fractions. Measurement sensitivity for known and indicated water vapour content was tested for one instrument, the error between wet and dry readings due to the instrument being uncalibrated for water vapour was only 0.24% ±0.04% for a 28% error in water vapour reading. Note that in our results only dry mole fractions are reported.

### 4.3. Closed-loop test uncertainty

With closed-loop operation, estimated loop volume may vary between blocks of test data, and running a triplicate standard for each set of tests in the mid-lower range of the instrument (e.g., 100 μl x 10,000 ppm CH_4_ for ca. 10 ppm ΔX) is advisable. The mean loop volume for the three instruments we use are 95.7, 98.8, and 114.6 ml (Table 1). The specific blocks of test data comprising the summary results presented in Table 1 are provided in the supplement (S1 Table). These were used to assess general accuracy (standard error) of the instruments. We combined all closed-loop injection test data for the 3 instruments covering the period June 2015 to May 2017. The range of standard gases used were 1000 to 100,000 ppm for CH_4_, and 5000 to 950,000 ppm CO_2_. The injection volume ranged from 20 μl to 1.5 ml, although the mean injection volume was 138 μl. The data include injections by at least 4 different instrument operators. Due to the differing standard gases, injection volumes, and resulting response concentrations, values were standardized to the expected concentration for each test group. The resultant overall CV for CH_4_ (n = 189) was 5.9%, and for CO_2_ (n = 114), 3.0% (Table 1). Due to the variability of the influencing factors we found no strong relationships between measurement error (ε) and expected equilibrium value, standard gas PP, or gas volume injected; although ε was more variable at lower than higher X_exp_. Syringe and operator error were investigated separately (see 4.5).

**Table 1:** Variability, accuracy of all closed-loop tests for three different instruments (F, R, and I) and for all instruments. X_meas_ and X_exp_ (in ppm) are the means of the measured and expected instrument PP, and V_loop_ gives estimated mean loop volume (ml) (SD and SE have the same units as their respective variables). Coefficient of variation CV(%) = 100(SD/X_exp_), standard error is SE = SD/√n.

ID	Variable	n	X_meas_	X_exp_	Mean (X_meas_/X_exp_)	†SD	SE	CV%
F	CH_4_ (ppm)	139	38.9	39.7	0.98	2.48	0.21	6.2
	CO_2_ (ppm)	93	997.1	1038.3	0.96	32.09	3.33	3.1
	V_loop_ (ml)	147	98.9			7.79	0.64	7.9
R	CH_4_ (ppm)	25	183.9	190.5	0.97	12.05	2.41	6.4
	CO_2_ (ppm)	3	499.2	498.5	1.00	0.58	0.33	0.1
	V_loop_ (ml)	28	95.7			6.18	1.17	6.5
I	CH_4_ (ppm)	24	27.1	27.3	0.99	0.94	0.19	3.4
	CO_2_ (ppm)	12	602.7	597.1	1.01	8.58	2.48	1.4
	V_loop_ (ml)	36	114.6			8.54	1.42	7.5
All	CH_4_ (ppm)	189	56.5	58.1	0.97	3.45	0.25	5.9
	CO_2_ (ppm)	114	942.8	974.8	0.97	28.64	2.68	3
	V_loop_ (ml)	208	101.2			3.75	0.26	9.5

^†^SD for CH_4_ and CO_2_ is calculated on the ratio of measured to expected values (to remove variation due to tests made with a range of standard gases and injection volumes) and then multiplied by the mean expected value (to scale back to PP).

### 4.4. Atmospheric pressure

In theory, the absolute effective loop volume is a function of the atmospheric pressure on the day of testing, whereas the laser chamber pressure is fixed. The physical volume of the partially evacuated system is approximately 380 ml, which at 18.7 kPa contains 70 ml of mixed gases. The mean estimated effective loop volume is 98.7 ml, therefore ca. 29 ml of the loop is at atmospheric pressure. Effective mixing volume should vary by -0.9% to +0.5% for pressure between 98.0 to 103.0 kPa, indeed, for atmospheric pressure 99.5 to 100.6 kPa, under near constant laboratory temperature, loop volume estimated from standard 100 μl injections of 5000/100000 ppm CH_4_/CO_2_ made over a number of weeks showed no consistent pattern (n = 29, R^2^ = 0.036). Thus measurement uncertainty due atmospheric pressure variation is effectively unmeasurable compared to other sources of error.

### 4.5. Sample injection

Operator handling of the syringe and injection practice can result in large measurement variation (see below), and to maximize precision and repeatability, great operator care is needed, e.g., SGE Analytical Science Co. [36] provide detailed advice on setting the syringe plunger to the same volume. To demonstrate potential operator and syringe variability, we compared injection repeatability with a gastight “syringe A” (1710RN, 100 μl, Hamilton, Switzerland) used for several years, a new regular (non-gastight) “syringe B” for liquid samples (710RN, 100 μl, Hamilton, Switzerland), and a new gas tight “syringe C” (1710RN, 100 μl, Hamilton, Switzerland). We injected 5 replicates of 100 μl of a dual standard (5.0x10^4^ ppm CH_4_ and 9.5x10^5^ ppm CO_2_) for each syringe and for 4 different operators (Table 2, supplement S3 Fig).

**Table 2:** Comparative injection measurement variability/error, for: A—Worn gastight syringe; B—New liquid syringe; and C—New gas tight syringe (see text for details), and 4 syringe operators (indicated by numbers 1 to 4). X_mean_ is the mean test gas PP in ppm. Expected test values were 50.1 ppm CH_4_ and 970.4 ppm CO_2_. Measurement error ε(%) is a given by Eq 3. CV is coefficient of variation (SD/X_mean_) standard error is SE = SD/√n, and SD is standard deviation.

	n	Gas	X_mean_ (ppm)	SD (ppm)	Min (ppm)	Max (ppm)	ε%	SE (ppm)	CV%
Comparison by syringe (all 4 operators)									
A	20	CH_4_	45.8	6.4	33.5	53.8	-9.4	1.4	13.9
		CO_2_	867.9	126.4	620.2	1017.8	-9.7	28.3	14.6
B	20	CH_4_	49.5	1.8	46.5	52.2	-2.2	0.4	3.5
		CO_2_	945.0	29.4	891.0	989.8	-1.7	6.6	3.1
C	20	CH_4_	49.3	1.4	47.4	53.0	-2.5	0.3	2.9
		CO_2_	928.1	31.5	884.3	997.9	-3.4	7.0	3.4
Comparison by operator (syringes B and C only)									
1	10	CH_4_	48.2	1.2	46.5	50.1	-4.6	0.4	2.5
		CO_2_	925.8	22.2	891.0	958.1	-3.7	7.0	2.4
2	10	CH_4_	48.7	0.8	47.4	50.4	-3.8	0.3	1.7
		CO_2_	925.3	20.6	892.2	967.1	-3.7	6.5	2.2
3	10	CH_4_	49.2	0.8	47.9	50.3	-2.7	0.3	1.7
		CO_2_	921.6	29.0	884.3	956.8	-4.1	9.2	3.1
4	10	CH_4_	51.5	0.9	49.6	53.0	1.9	0.3	1.7
		CO_2_	973.5	21.0	924.4	997.9	1.3	6.6	2.2

^†^Between operator data excludes old syringe A, hence n = 10, not 15.

The specific syringe measurement CV for CH_4_ and CO_2_ were ±3.6 and ±3.1% for the liquid syringe (Table 2B), ±2.9 and ±3.4% for the new gastight syringe (C) and between 13.9 and 14.6% for the worn syringe (A) (n = 20 for each syringe, including data for the 4 operators). This was greater than variations between operators (where data for the worn syringe (A) were excluded); around ±1.7% for CH_4_ (operators 2 to 4), ±2.4% for operator 1 (mean for 4 operators 1.9 for CH_4_, and 2.48 for CO_2_). The absolute measurement error was around -4% (under-reading) for operators 1 to 3, but for operator 4 around +1.5% (over-reading, >5% more than other operators when apparently carrying-out the same injection practice). The worn syringe (A) presented a worst case with large underestimates and high variability (see S3 Fig in supplement), although operator 4, who achieved a positive absolute error in all tests, had CV for syringe A comparable to those for the other operators for syringe B and C., and also achieved an over-reading (see S2 Table and S3 Fig for more detailed data).

The speed at which the sample gas is drawn into the syringe and then injected into the closed-loop may cause additional measurement variability, and also relates to syringe condition and quality of maintenance. However, this error is a combination of the injection repeatability error mentioned above, and the error related to the speed of drawing/injecting the gas, the two errors are not distinguishable. Our tests (*n* = 10 for CH_4_ and CO_2_, respectively) revealed that “slow” injections (full depression of syringe piston in ca. 2 sec) resulted in 4.3 ± 5.6% (average ±1 SD) higher ΔX for CH_4_ and 3.3 ± 6.0% higher ΔX for CO_2_ compared to fast injections (the syringe piston depressed rapidly, ca. 0.5 s). Operator and syringe-related measurement errors can probably be minimized by using an automated injection system if this suits the repetitiveness of the particular measurement application.

### 4.6. Stacked sample injection

Stacked sampling procedures were investigated, where samples were injected one after another without venting (see S5 Fig). Rapid testing could be achieved, but at a cost to accuracy because of a sharp drop-off in measured compared to expected values with successive sample injections. The error (ε %, Eq 3) on the equilibrium PP values generally increased with the number of samples injected. At around 100 ppm CH_4_, and 2500 ppm CO_2_, ε ranged from -7 to -18%, and -5 to -13% (for injection groups resulting in total X_CH4_ ≥ 100 ppm; 12 x 20 μl, 6 x 40 μl, 4 x 60 μl, 3 x 100 μl of 50000/200000 ppm CH_4_/CO_2_ in nitrogen standard, respectively, vented between each group injections). The error on the stacked ΔX values (S5 Fig) was around 70% greater than for the total measured X_CH4_ and X_C02_ equilibrium values. To adjust X_exp_ and ΔX_exp_ we corrected for increasing dilution due to the total increase in loop volume (the diluting mass of gas in the loop) resulting from the summed injected volumes, leakage between injections, water vapour variation, or syringe under-sampling, but the total measurement drop-off could not be accounted for. With a succession of real samples, their PP values may vary over a wide range, consequently, the ΔX error is irregular making attempts to correct imprecise and unrepeatable, and thus we cannot recommended sample stacking at the time of writing.

## 5. Data processing

We improved our operational efficiency with the UGGA system in closed-loop configuration by minimizing manual data reading and recording, and automating sample detection from the raw instrument output data. The calculation of final PP of CO_2_ and CH_4_ using Eq (1) requires values of both PP in the gas loop before and after sample injection (i.e. to get the ΔX values; see Fig 3a), and the sample ID and instrument time must be recorded.

### 5.1. Manual data reading

PP values can be manually noted from the UGGA display during the measurement. Manually reading and writing-out of the values (with associated sample Id and time of reading) is repeated four times per sample (for both CH_4_ and CO_2_), and may comprise the majority of the time spent at the instrument; one sample test with data reading and recording takes a minimum of three to five minutes. In addition to the cost in time, operator data reading and recording inconsistencies may be introduced. Accurate manual reading of values from the instrument display is hampered by the fluctuation of displayed values, and where values are extracted by zooming into the graphical display operator practices may differ. Further data recording uncertainties may arise from inconsistent rounding of decimal places, and careless recording of values in log books, and subsequent data input (digitization) errors.

### 5.2. Automated data extraction

To reduce sample operation time and to minimize potential operator inconsistencies, we created a spreadsheet based tool for extracting the measured PP and calculating the sample values of CO_2_ and CH_4_ (supplement S2_extractor.xlsb). The algorithms in the analysis package smooth the data (based on the approach presented in Wilkinson et al.,[28]) and identify each sample injection point, then calculate mean values for the baseline and equilibrium sample values from consistent representative parts of the data for every measurement (S1 Fig).

With the data extractor tool, raw data are taken from the UGGA and loaded into the “raw data” tab of the extractor, the data are visualized (plotting tab, see S1 Fig), and sample peak capture can be optimized by adjusting step-threshold, and smoothing parameters (plotting tab). The extractor lists each test result in sequence (results tab, see S2 Fig), and calculates the sample concentration in ppm (Eq 1), μg/l, and injected mass (ng), and the equivalent value in moles per litre, or mg per litre, these can be checked against the sample IDs and times and copied into a new sheet for further analysis. In addition, the extractor provides the ring down for each laser in the plotting tab. The same sample injection order should be used for regular repeat batch testing, this eliminates the need for data sorting and rearrangement when comparing sub-sequent tests; only sample times and total batch sample number have to be logged.

Automated data recovery from raw data files in post-processing reduced our instrument time by a factor of three to five, this was particularly helpful when processing large sample numbers. Typically one sample measurement takes 40 seconds to 2 minutes (Fig 3a). The time-limiting steps are sample extraction and injection (10 s), plus instrument response (rise and equilibrium 15–30 s), and sample venting (45 s including writing), in total around 70 to 95 s with a practiced operator. Only the start time of each injection and the sample ID has to be recorded.

## 6. Conclusions and recommendations

With the increasing use of portable greenhouse gas analyzers, and the need to test large numbers of samples quickly, at low cost, and often in the field, and sometimes in difficult or remote locations, the adaption of such analyzers for closed-loop operation significantly extends their utility. We adapted one type of PGA instrument for closed-loop operation and investigated measurement range, calibration and maintenance, accuracy and efficiency issues. Closed-loop CH_4_ and CO_2_ partial pressure in small-volume gas samples (100 μl) could be reliably measured for samples ranging between 4.5 ppm and 9 x10^4^ ppm (CH_4_) and 1000 ppm to 1 x10^6^ ppm (CO_2_) with an average total measurement uncertainty (SE) of ±5.9% and ±3.0%, respectively. Errors and adjustments for water vapour calibration (0.25% for a 28% error in water vapour measurement), and atmospheric pressure variation (-0.93% to +0.48% for 98.0 to 103.0 kPa) were small. Open-loop response non-linearity for CH_4_ above the nominal measurement range (100 ppm; -10.3% and -4.9% at 300 ppm) was observed in two of the three PGAs tested; and a calibration curve should be determined if high CH_4_ values are routinely expected to be measured. CO_2_ response remained linear for a 950 ppt injected standard.

To ensure on-going accurate measurement and reliable operation, the following points are made: A well-sealed system is ideal for PGA use in closed-loop—leakage is evident from a steady decline in concentration after a test injection, and may be due to loose tubing joints, pump diaphragm deterioration, and (rarely) mirror edge damage. Loop volume calculation for each testing batch is strongly encouraged (triplicate injected methane standard for readings in the mid-lower range, i.e., 10–50 ppm). Routine recording of ring down time (where applicable) (as in our data extractor tool) highlights when mirror cleaning is needed (RD <6 μs). Finally, regular (e.g., monthly) open-loop calibration checks are recommended.

The main source of measurement uncertainty (coefficient of variation from repeated measurement) was external to the instrument, and related to sample injection—operator practices and syringe condition. Operator induced under-reading was more common than over-reading, but was consistent from operator to operator. CV with well-maintained syringes operated by different individuals was around 3%, however, individual operators generally achieved lower CV (ca. 1.7% for CH_4_). In contrast an old worn syringe showed potential for large under-readings (> -9%) and high variability (CV = 14%). The speed of syringe piston movement was important; slow syringe operation was found deliver 3 to 4% more sample gas on average, than with rapid movement. In general, the results highlight the need for consistent syringe operating practices and good maintenance of syringes.

Finally, we achieved a factor of 3 to 5-fold improvement in operational efficiency by automating data recovery. This reduced time at the instrument (per sample processing time < 2 min), time taken writing and digitizing, and standardized recovery of measured values, thus eliminating errors from manual reading and recording, as well as, accelerating calculation and reporting activities.

## Supporting information

S1 Fig Data extractor tool “plotting” tab. Shows visualization of processed data with marked mean baseline and equilibrium gas PP, laser ring down values, smoothing and step detection parameters.
(TIF)

S2 Fig Data extractor tool “results” tab. Shows ΔX values and calculated original sample values. Closed-loop volume and injection volume is entered here. Columns with baseline and equilibrium means are not shown in this screenshot.
(TIF)

S3 Fig Comparison of errors for syringe and operator repeatability testing, arranged by syringe and operator (see also S2 Table). Percentage error is (X_meas_-X_exp_)/X_exp_*100%. Syringe A presents a worst case, and operator 4 achieved much closer replicates and higher values than the other operators.
(TIF)

S4 Fig Extreme ring-down reduction—Effect of accidental water ingress on ring down time (RD) for background noise for CH_4_ and CO_2_ detectors; a. and c. clean mirrors (RD > 9 μs), b. and d. after drying of loop without mirror cleaning (RD < 4 μs). (TIF)

S5 Fig Example stacked injection data for sediment incubation headspace gases for paired flasks of sub-samples from increasing depths below surface water-sediment interface. (TIF)

S1 Table Summary statistics for collected closed-loop injection tests between 5 June 2015 and 17 May 2017. The quoted mean values are composed of data that may span a range of test gas concentrations (ppm). The loop volume was the average estimated loop volume based on all values for each date group. X_meas_ and X_exp_ (in ppm) are the means of the measured and expected instrument PP, and V_loop_ gives estimated mean loop volume (ml) (SD and SE have the same units as their respective variables). Coefficient of variation CV(%) = 100(SD/X_mean_), standard error is SE = SD/√n.
(DOCX)

S2 Table Detailed statistics for syringe and operator injection tests as presented in S3 Fig. For: A—worn gastight syringe; B—new liquid syringe; and C—new gas tight syringe (see text for details), and 4 syringe operators (indicated by numbers 1 to 4). X_mean_ is the mean test gas PP in ppm. Expected test values were 50.1 ppm CH_4_ and 970.4 ppm CO_2_. Measurement error ε(%) is given by Eq 3. CV is coefficient of variation (SD/X_mean_) standard error is SE = SD/√n, and SD is standard deviation.
(DOCX)

S1 File S1_File.pdf. (PDF)

S2 File S2_extractor.xlsb. (XLSB)

S3 File Collected-figure-data.xlxs. (XLSX)
