# Correction: Measuring CO_2_ and CH_4_ with a portable gas analyzer: Closed-loop operation, optimization and assessment

**DOI:** 10.1371/journal.pone.0206080

**Published:** 2019-03-06

**Authors:** Jeremy Wilkinson, Christoph Bors, Florian Burgis, Andreas Lorke, Pascal Bodmer

There are errors in S2 File. Please view the correct S2 File below.

There are errors in Eqs 1, 2 and 4. Please view the complete, correct equations here:

$$X_{s}=(\frac{V_{l}+V_{s}}{V_{s}})\Delta X+X_{0}=\frac{V_{l}}{V_{s}}\Delta X+X_{\infty }$$ (1)

V_s_ is the injected sample volume, V_l_ is loop volume, and X_∞_ is the steady final plateau concentration, and ΔX = X_∞_ − X_0_.

$$V_{l}=(\frac{X_{s}-X_{0}}{\Delta X})V_{s}-V_{s}$$ (2)

$$X_{\infty }=\frac{X_{s}V_{s}+{X_{0}V}_{l}}{V_{s}+V_{l}}=X_{exp}=\frac{X_{stn}V_{s}+{X_{0}V}_{l}}{V_{s}+V_{l}}$$ (4)

X_exp_ is the expected X_∞_ (i.e. the steady final plateau concentration), and X_stn_ is the standard (sample) gas concentration of the injected Xs.

## Supporting information

S2 File S2_extractor.xlsb. (XLSB)
