# Supplementary figures and images for: Measuring CO2 and CH4 with a portable gas analyzer: Closed-loop operation, optimization and assessment
Source: PLoS One. 2018 Apr 4;13(4):e0193973. doi: 10.1371/journal.pone.0193973 (PMC5884480; doi:10.1371/journal.pone.0193973)

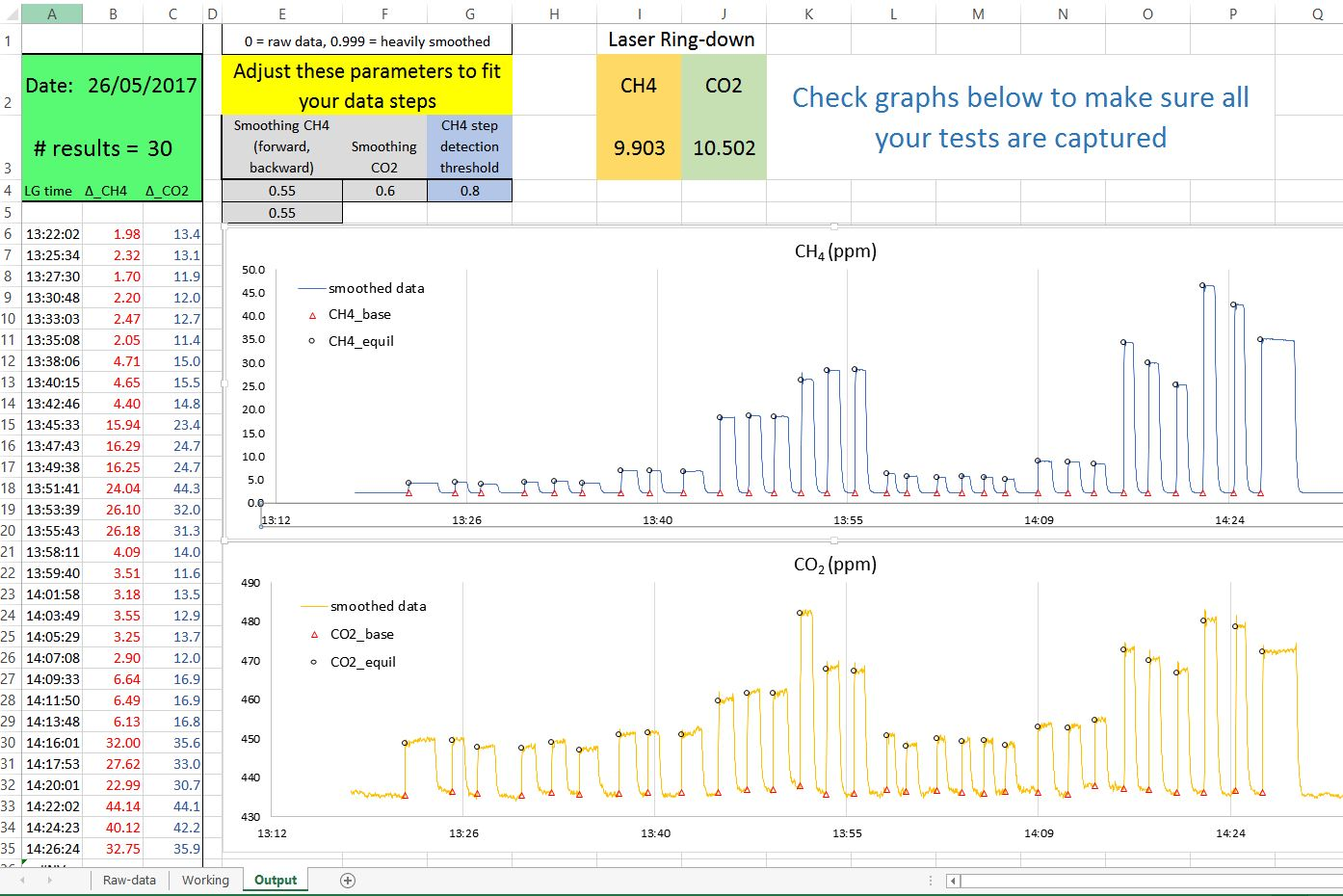

Supplement: S1 Fig — Shows visualization of processed data with marked mean baseline and equilibrium gas PP, laser ring down values, smoothing and step detection parameters. (TIF) [file pone.0193973.s001.tif]

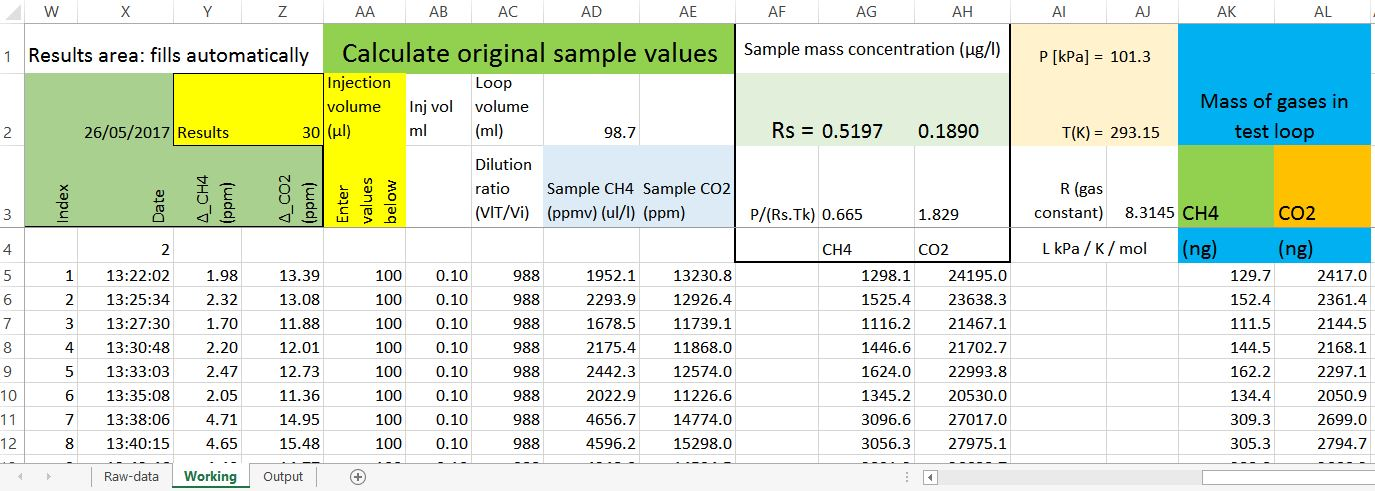

Supplement: S2 Fig — Shows ΔX values and calculated original sample values. Closed-loop volume and injection volume is entered here. Columns with baseline and equilibrium means are not shown in this screenshot. (TIF) [file pone.0193973.s002.tif]

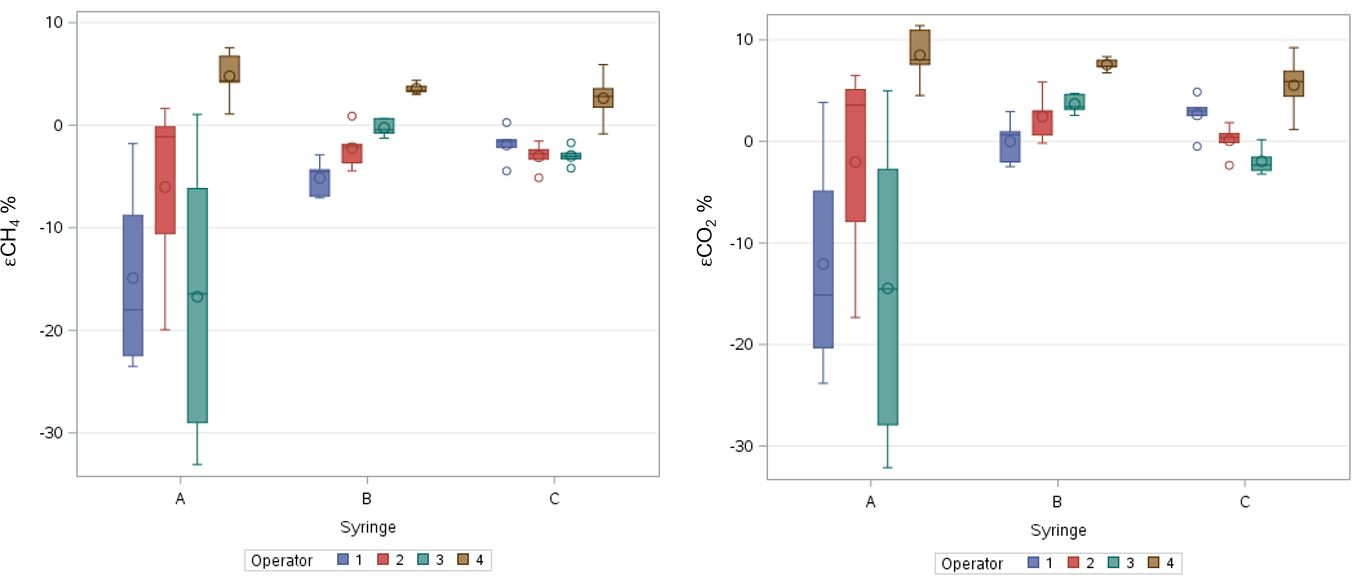

Supplement: S3 Fig — Percentage error is (Xmeas-Xexp)/Xexp*100%. Syringe A presents a worst case, and operator 4 achieved much closer replicates and higher values than the other operators. (TIF) [file pone.0193973.s003.tif]

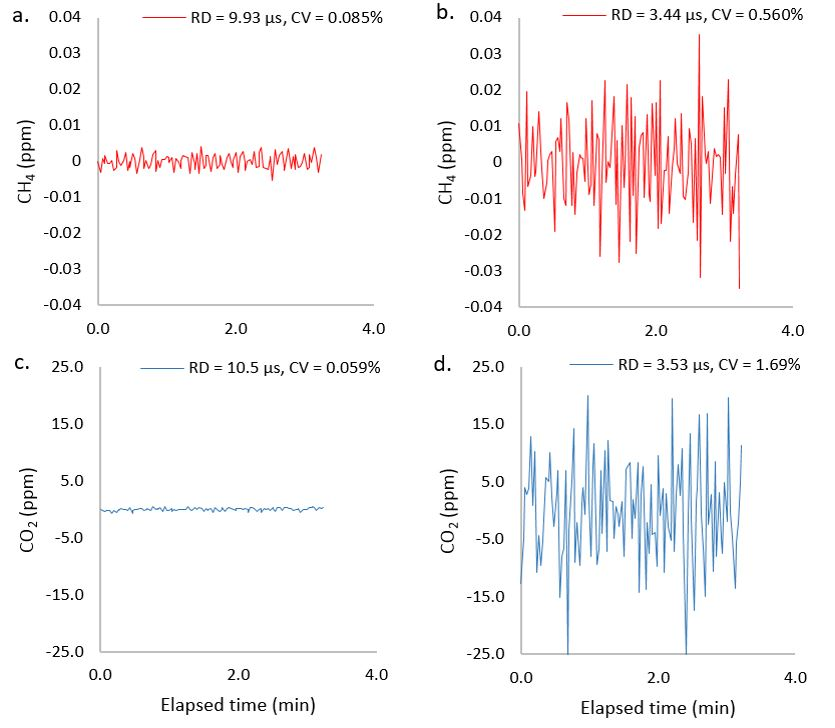

Supplement: S4 Fig — (TIF) [file pone.0193973.s004.tif]

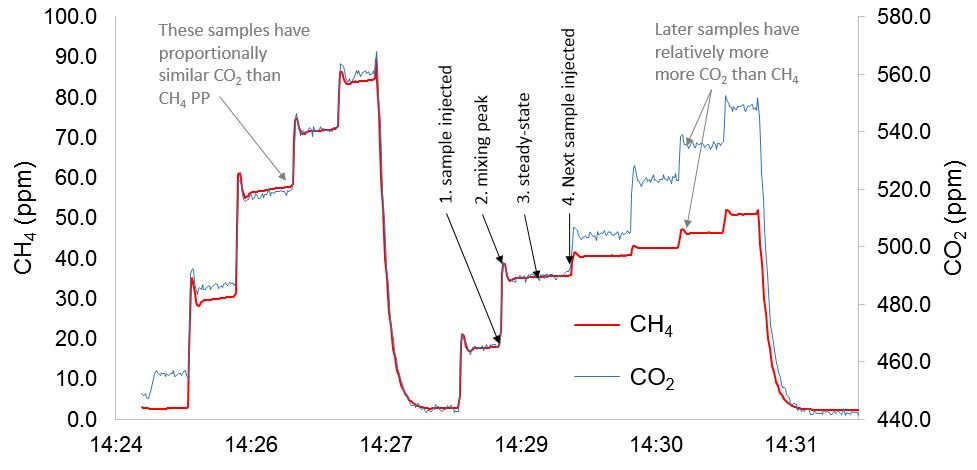

Supplement: S5 Fig — (TIF) [file pone.0193973.s005.tif]
