# Supplementary material for: Measuring CO2 and CH4 with a portable gas analyzer: Closed-loop operation, optimization and assessment
Source: PLoS One. 2018 Apr 4;13(4):e0193973. doi: 10.1371/journal.pone.0193973 (PMC5884480; doi:10.1371/journal.pone.0193973)
Supplement: S1 Table — The quoted mean values are composed of data that may span a range of test gas concentrations (ppm). The loop volume was the average estimated loop volume based on all values for each date group. Xmeas and Xexp (in ppm) are the means of the measured and expected instrument PP, and Vloop gives estimated mean loop volume (ml) (SD and SE have the same units as their respective variables). Coefficient of variation CV(%) = 100(SD/Xmean), standard error is SE = SD/√n. (DOCX) [file pone.0193973.s006.docx]

| ID | Date | Variable | X_obs_ | X_exp_ | mean (obs/ exp) | Std Dev | n | SE | CV% | Std Dev | ±SE% |
| --- | --- | --- | --- | --- | --- | --- | --- | --- | --- | --- | --- |
| F | 5/06/2015 | CH_4_ | 6.3 | 6.4 | 1.01 | 0.43 | 7 | 0.16 | 6.63 | 0.07 | 0.03 |
|  |  | Vloop | 94.1 |  |  | 11.78 | 7 | 4.45 | 12.52 | 11.78 | 4.45 |
|  | 29/10/2015 | CH_4_ | 8.0 | 7.9 | 1.01 | 0.37 | 22 | 0.08 | 4.67 | 0.05 | 0.01 |
|  |  | Vloop | 98.9 |  |  | 7.06 | 22 | 1.51 | 7.14 | 7.06 | 1.51 |
|  | 7/12/2015 | CH_4_ | 3.2 | 3.2 | 1.00 | 0.02 | 5 | 0.01 | 0.51 | 0.01 | 0.00 |
|  |  | CO_2_ | 468.5 | 469.5 | 1.00 | 1.61 | 6 | 0.66 | 0.34 | 0.00 | 0.00 |
|  |  | Vloop | 101.8 |  |  | 4.15 | 7 | 1.57 | 4.08 | 4.15 | 1.57 |
|  | 9/12/2015 | CH_4_ | 8.8 | 8.5 | 1.02 | 0.13 | 5 | 0.06 | 1.45 | 0.01 | 0.01 |
|  |  | CO_2_ | 588.4 | 586.3 | 1.00 | 3.19 | 4 | 1.59 | 0.54 | 0.01 | 0.00 |
|  |  | Vloop | 85.4 |  |  | 2.31 | 5 | 1.03 | 2.71 | 2.31 | 1.03 |
|  | 13/03/2017 | CH_4_ | 22.8 | 22.9 | 1.00 | 0.96 | 6 | 0.39 | 4.23 | 0.04 | 0.02 |
|  |  | CO_2_ | 472.0 | 471.6 | 1.00 | 0.69 | 7 | 0.26 | 0.15 | 0.00 | 0.00 |
|  |  | Vloop | 96.7 |  |  | 5.06 | 12 | 1.46 | 5.23 | 5.06 | 1.46 |
|  | 24/04/2017 | CH_4_ | 128.7 | 134.2 | 1.02 | 13.79 | 13 | 3.82 | 10.12 | 0.10 | 0.03 |
|  |  | Vloop | 99.9 |  |  | 6.29 | 12 | 1.82 | 6.29 | 6.29 | 1.82 |
|  | 9/05/2017 | CH_4_ | 52.9 | 53.3 | 0.99 | 1.54 | 43 | 0.23 | 2.90 | 0.03 | 0.00 |
|  |  | CO_2_ | 1403.2 | 1412.9 | 0.99 | 30.99 | 43 | 4.73 | 2.21 | 0.02 | 0.00 |
|  |  | Vloop | 100.0 |  |  | 3.26 | 43 | 0.50 | 3.26 | 3.26 | 0.50 |
|  | 17/05/2017 | CH_4_ | 27.3 | 28.2 | 1.00 | 2.45 | 38 | 0.40 | 8.69 | 0.09 | 0.01 |
|  |  | CO_2_ | 760.7 | 828.5 | 0.99 | 37.47 | 33 | 6.52 | 4.58 | 0.05 | 0.01 |
|  |  | Vloop | 99.7 |  |  | 10.81 | 38 | 1.75 | 10.84 | 10.81 | 1.75 |
| ID | Date | Variable | X_obs_ | X_exp_ | mean (obs/ exp) | Std Dev | n | SE | CV% | Std Dev | ±SE% |
| I | 12/10/2016 | CH_4_ | 27.1 | 27.3 | 1.00 | 0.94 | 24 | 0.19 | 3.43 | 0.03 | 0.01 |
|  |  | Vloop | 116.2 |  |  | 9.22 | 24 | 1.88 | 7.94 | 9.22 | 1.88 |
|  | 8/11/2016 | CO_2_ | 602.7 | 597.1 | 1.01 | 8.58 | 12 | 2.48 | 1.42 | 0.01 | 0.00 |
|  |  | Vloop | 111.4 |  |  | 6.16 | 12 | 1.78 | 5.53 | 6.16 | 1.78 |
| R | 7/12/2015 | CH_4_ | 8.0 | 8.0 | 1.00 | 0.00 | 2 | 0.00 | 0.00 | 0.00 | 0.00 |
|  |  | Vloop | 86.5 |  |  | 0.01 | 2 | 0.01 | 0.01 | 0.01 | 0.01 |
|  | 9/12/2015 | CH_4_ | 31.6 | 28.1 | 1.12 | 5.15 | 2 | 3.64 | 16.32 | 0.18 | 0.13 |
|  |  | Vloop | 97.0 |  |  | 15.09 | 2 | 10.67 | 17.48 | 15.09 | 10.67 |
|  | 27/03/2017 | CH_4_ | 106.9 | 106.8 | 1.00 | 1.83 | 3 | 1.06 | 1.71 | 0.02 | 0.01 |
|  |  | CO_2_ | 499.2 | 498.5 | 1.00 | 0.58 | 3 | 0.33 | 0.12 | 0.00 | 0.00 |
|  |  | Vloop | 93.8 |  |  | 3.75 | 6 | 1.53 | 4.00 | 3.75 | 1.53 |
|  | 7/04/2017 | CH_4_ | 233.3 | 242.8 | 0.97 | 8.72 | 18 | 2.06 | 3.69 | 0.04 | 0.01 |
|  |  | Vloop | 98.4 |  |  | 3.67 | 18 | 0.87 | 3.73 | 3.67 | 0.87 |
