# Supplementary material for: Measuring CO2 and CH4 with a portable gas analyzer: Closed-loop operation, optimization and assessment
Source: PLoS One. 2018 Apr 4;13(4):e0193973. doi: 10.1371/journal.pone.0193973 (PMC5884480; doi:10.1371/journal.pone.0193973)
Supplement: S2 Table — For: A—worn gastight syringe; B—new liquid syringe; and C—new gas tight syringe (see text for details), and 4 syringe operators (indicated by numbers 1 to 4). Xmean is the mean test gas PP in ppm. Expected test values were 50.1 ppm CH4 and 970.4 ppm CO2. Measurement error ε(%) is given by Eq 3. CV is coefficient of variation (SD/Xmean) standard error is SE = SD/√n, and SD is standard deviation. (DOCX) [file pone.0193973.s007.docx]

| Operator | Syringe | n | Gas (ppm) | X_mean_ | SD | X_min_ | X_max_ | ε(%) | SE | CV(%) |
| --- | --- | --- | --- | --- | --- | --- | --- | --- | --- | --- |
| 1 | A | 5 | CH_4_ | 42.3 | 5 | 36.9 | 49.1 | -16.4 | 2.2 | 11.9 |
|  |  |  | CO_2_ | 803.4 | 104.2 | 696 | 948.7 | -16.4 | 46.6 | 13 |
|  | B | 5 | CH_4_ | 47.4 | 0.9 | 46.5 | 48.6 | -6.2 | 0.4 | 1.9 |
|  |  |  | CO_2_ | 913.7 | 20.5 | 891 | 940.4 | -4.9 | 9.2 | 2.2 |
|  | C | 5 | CH_4_ | 49.1 | 0.8 | 47.8 | 50.1 | -3 | 0.4 | 1.7 |
|  |  |  | CO_2_ | 937.9 | 17.9 | 909.2 | 958.1 | -2.4 | 8 | 1.9 |
| 2 | A | 5 | CH_4_ | 47 | 4.6 | 40 | 50.8 | -7.1 | 2 | 9.7 |
|  |  |  | CO_2_ | 895.3 | 94 | 755.2 | 972.9 | -6.8 | 42 | 10.5 |
|  | B | 5 | CH_4_ | 48.9 | 1 | 47.8 | 50.5 | -3.4 | 0.5 | 2.1 |
|  |  |  | CO_2_ | 936 | 21.6 | 912.1 | 967.1 | -2.6 | 9.6 | 2.3 |
|  | C | 5 | CH_4_ | 48.5 | 0.7 | 47.4 | 49.2 | -4.1 | 0.3 | 1.4 |
|  |  |  | CO_2_ | 914.6 | 14.2 | 892.2 | 930.6 | -4.8 | 6.3 | 1.5 |
| 3 | A | 5 | CH_4_ | 41.6 | 7.3 | 33.5 | 50.5 | -17.7 | 3.3 | 17.5 |
|  |  |  | CO_2_ | 781.5 | 145 | 620.2 | 959.2 | -18.7 | 64.9 | 18.6 |
|  | B | 5 | CH_4_ | 49.9 | 0.4 | 49.4 | 50.3 | -1.4 | 0.2 | 0.9 |
|  |  |  | CO_2_ | 947.4 | 8.6 | 937.1 | 956.8 | -1.4 | 3.8 | 0.9 |
|  | C | 5 | CH_4_ | 48.5 | 0.4 | 47.9 | 49.1 | -4.1 | 0.2 | 0.9 |
|  |  |  | CO_2_ | 895.8 | 12.2 | 884.3 | 915.2 | -6.8 | 5.5 | 1.4 |
| 4 | A | 5 | CH_4_ | 52.4 | 1.3 | 50.5 | 53.8 | 3.6 | 0.6 | 2.4 |
|  |  |  | CO_2_ | 991.3 | 25.5 | 955 | 1017.8 | 3.1 | 11.4 | 2.6 |
|  | B | 5 | CH_4_ | 51.8 | 0.3 | 51.5 | 52.2 | 2.4 | 0.1 | 0.5 |
|  |  |  | CO_2_ | 982.7 | 5.6 | 975.3 | 989.8 | 2.3 | 2.5 | 0.6 |
|  | C | 5 | CH_4_ | 51.3 | 1.2 | 49.6 | 53 | 1.5 | 0.6 | 2.4 |
|  |  |  | CO_2_ | 964.2 | 27.3 | 924.4 | 997.9 | 0.3 | 12.2 | 2.8 |
| All | A | 20 | CH_4_ | 45.8 | 6.4 | 33.5 | 53.8 | -9.4 | 1.4 | 13.9 |
|  |  |  | CO_2_ | 867.9 | 126.4 | 620.2 | 1017.8 | -9.7 | 28.3 | 14.6 |
|  | B | 20 | CH_4_ | 49.5 | 1.8 | 46.5 | 52.2 | -2.2 | 0.4 | 3.5 |
|  |  |  | CO_2_ | 945 | 29.4 | 891 | 989.8 | -1.7 | 6.6 | 3.1 |
|  | C | 20 | CH_4_ | 49.3 | 1.4 | 47.4 | 53 | -2.5 | 0.3 | 2.9 |
|  |  |  | CO_2_ | 928.1 | 31.5 | 884.3 | 997.9 | -3.4 | 7 | 3.4 |
