# Supplementary material for: Measuring CO2 and CH4 with a portable gas analyzer: Closed-loop operation, optimization and assessment
Source: PLoS One. 2018 Apr 4;13(4):e0193973. doi: 10.1371/journal.pone.0193973 (PMC5884480; doi:10.1371/journal.pone.0193973)
Supplement: S1 File — (PDF) [file pone.0193973.s008.pdf]

Supplementary information: Measuring CO2 and CH4 with a portable gas analyzer: Closed-loop operation, optimization and assessment

S1. Further details on the Data Extractor (spreadsheet template to be attached with example data, see also S1 Fig and S2 Fig, below)

Data capture algorithms, both linear and log_{10} transformed data were used during the development of the extractor, eventually log_{10} transformed data were used because this amplified the lower value data making lower concentration response steps easier to identify. In addition, the extractor applies smoothing (as described in [1]) to damp the initial circulation peak (Fig. 3 main text), and any subsequent excessive noise. The extractor tests the $log_{10}[CH_4]$ time-series for step increases in concentration of a minimum magnitude, and for each detection takes the mean of the smoothed non-transformed 5 values preceding and following the step increase. Timings for CO_2 data extraction, follow those for CH_4, this has the minor disadvantage that if no CH_4 is present in a sample the CO_2 peak is missed (if this is a common problem, column B and column C in the results tab can be reassigned to look-up CO_2 and then CH_4 in the raw data). The ability to adjust the detection parameters in real-time has certain advantages over script based post-processing, since the operator can optimize the sample detection immediately without re-running a script. The base line and equilibrium values for both gases are recorded in the results table with the relevant time, and the points are marked in the graphics. In addition, the sample partial pressure is calculated based-on the indicated instrument loop volume. The exact timing of the pre and post-response means has been adjusted to avoid the initial rise, and the roll-over to the equilibrium value, thus providing the best estimate of the instrument response to the injected sample. The difference between the baseline and equilibrium values (ΔX) is computed and logged in the results. The step detection threshold can be adjusted to suit high or low concentration data, and the smoothing

varied if necessary, generally fixed smoothing parameters are adequate, although adjustment may be required to get the best results with low or high concentration data blocks.

S1 Fig. Data extractor tool “plotting” tab. Shows visualization of processed data with marked mean baseline and equilibrium gas PP, laser ring down values, smoothing and step detection parameters.

S2 Fig. Data extractor tool “results” tab. Shows ΔX values and calculated original sample values. Closed-loop volume and injection volume is entered here. Columns with baseline and equilibrium means are not shown in this screenshot.

S1 Table. Summary statistics for collected closed-loop injection tests between 5 June 2015 and 17 May 2017. The quoted mean values are composed of data that may span a range of test gas concentrations (ppm). The loop volume was the average estimated loop volume based on all values for each date group. X_{meas} and X_{exp} (in ppm) are the means of the measured and expected instrument PP, and V_{loop} gives estimated mean loop volume (ml) (SD and SE have the same units as their respective variables). Coefficient of variation CV(%)=100(SD/X_{mean}), standard error is SE = SD/√n.

ID	Date	N	Variable	X_{meas}	X_{exp}	mean (obs/ exp)	†SD	n	SE	CV%
F	05/06/2015	7	CH_4 (ppm)	6.3	6.4	1.01	0.43	7	0.16	6.63
			V_{loop} (ml)	94.1			11.78	7	4.45	12.52
	29/10/2015	22	CH_4 (ppm)	8.0	7.9	1.01	0.37	22	0.08	4.67
			V_{loop} (ml)	98.9			7.06	22	1.51	7.14
	07/12/2015	7	CH_4 (ppm)	3.2	3.2	1.00	0.02	5	0.01	0.51
			CO_2 (ppm)	468.5	469.5	1.00	1.61	6	0.66	0.34
			V_{loop} (ml)	101.8			4.15	7	1.57	4.08
	09/12/2015	5	CH_4 (ppm)	8.8	8.5	1.02	0.13	5	0.06	1.45
			CO_2 (ppm)	588.4	586.3	1.00	3.19	4	1.59	0.54
			V_{loop} (ml)	85.4			2.31	5	1.03	2.71
	13/03/2017	13	CH_4 (ppm)	22.8	22.9	1.00	0.96	6	0.39	4.23
			CO_2 (ppm)	472.0	471.6	1.00	0.69	7	0.26	0.15
			V_{loop} (ml)	96.7			5.06	12	1.46	5.23
	24/04/2017	13	CH_4 (ppm)	128.7	134.2	1.02	13.79	13	3.82	10.12
			V_{loop} (ml)	99.9			6.29	12	1.82	6.29
	09/05/2017	43	CH_4 (ppm)	52.9	53.3	0.99	1.54	43	0.23	2.90
			CO_2 (ppm)	1403.2	1412.9	0.99	30.99	43	4.73	2.21
			V_{loop} (ml)	100.0			3.26	43	0.50	3.26
	17/05/2017	38	CH_4 (ppm)	27.3	28.2	1.00	2.45	38	0.40	8.69
			CO_2 (ppm)	760.7	828.5	0.99	37.47	33	6.52	4.58
			V_{loop} (ml)	99.7			10.81	38	1.75	10.84
I	12/10/2016	24	CH_4 (ppm)	27.1	27.3	1.00	0.94	24	0.19	3.43
			V_{loop} (ml)	116.2			9.22	24	1.88	7.94
	08/11/2016	12	CO_2 (ppm)	602.7	597.1	1.01	8.58	12	2.48	1.42
			V_{loop} (ml)	111.4			6.16	12	1.78	5.53
R	07/12/2015	2	CH_4 (ppm)	8.0	8.0	1.00	0.00	2	0.00	0.00
			V_{loop} (ml)	86.5			0.01	2	0.01	0.01
	09/12/2015	2	CH_4 (ppm)	31.6	28.1	1.12	5.15	2	3.64	16.32
			V_{loop} (ml)	97.0			15.09	2	10.67	17.48
	27/03/2017	6	CH_4 (ppm)	106.9	106.8	1.00	1.83	3	1.06	1.71
			CO_2 (ppm)	499.2	498.5	1.00	0.58	3	0.33	0.12
			V_{loop} (ml)	93.8			3.75	6	1.53	4.00
	07/04/2017	18	CH_4 (ppm)	233.3	242.8	0.97	8.72	18	2.06	3.69
			V_{loop} (ml)	98.4			3.67	18	0.87	3.73

†SD for CH_4 and CO_2 is calculated on the ratio of measured to expected values (to remove variation due to tests made with a range of standard gases and injection volumes) and then multiplied by the mean expected value (to scale back to PP).

Table S2. Detailed statistics for syringe and operator injection tests as presented in Fig. S3 for: A – worn gastight syringe; B – new liquid syringe; and C - new gas tight syringe (see text for details), and 4 syringe operators (indicated by numbers 1 to 4). X_{mean} is the mean test gas PP in ppm. Expected test values were 50.1 ppm CH_4 and 970.4 ppm CO_2. Measurement error ε(%) is a given by Eq. 3. CV is coefficient of variation (SD/X_{mean}) standard error is $SE = SD/\sqrt{n}$, and SD is standard deviation.

Operator	Syringe	n	Gas	X_{mean} (ppm)	SD (ppm)	X_{min} (ppm)	X_{max} (ppm)	ε(%)	SE (ppm)	CV(%)
1	A	5	CH_4	42.3	5.0	36.9	49.1	-16.4	2.2	11.9
			CO_2	803.4	104.2	696.0	948.7	-16.4	46.6	13.0
	B	5	CH_4	47.4	0.9	46.5	48.6	-6.2	0.4	1.9
			CO_2	913.7	20.5	891.0	940.4	-4.9	9.2	2.2
	C	5	CH_4	49.1	0.8	47.8	50.1	-3.0	0.4	1.7
			CO_2	937.9	17.9	909.2	958.1	-2.4	8.0	1.9
2	A	5	CH_4	47.0	4.6	40.0	50.8	-7.1	2.0	9.7
			CO_2	895.3	94.0	755.2	972.9	-6.8	42.0	10.5
	B	5	CH_4	48.9	1.0	47.8	50.5	-3.4	0.5	2.1
			CO_2	936.0	21.6	912.1	967.1	-2.6	9.6	2.3
	C	5	CH_4	48.5	0.7	47.4	49.2	-4.1	0.3	1.4
			CO_2	914.6	14.2	892.2	930.6	-4.8	6.3	1.5
3	A	5	CH_4	41.6	7.3	33.5	50.5	-17.7	3.3	17.5
			CO_2	781.5	145.0	620.2	959.2	-18.7	64.9	18.6
	B	5	CH_4	49.9	0.4	49.4	50.3	-1.4	0.2	0.9
			CO_2	947.4	8.6	937.1	956.8	-1.4	3.8	0.9
	C	5	CH_4	48.5	0.4	47.9	49.1	-4.1	0.2	0.9
			CO_2	895.8	12.2	884.3	915.2	-6.8	5.5	1.4
4	A	5	CH_4	52.4	1.3	50.5	53.8	3.6	0.6	2.4
			CO_2	991.3	25.5	955.0	1017.8	3.1	11.4	2.6
	B	5	CH_4	51.8	0.3	51.5	52.2	2.4	0.1	0.5
			CO_2	982.7	5.6	975.3	989.8	2.3	2.5	0.6
	C	5	CH_4	51.3	1.2	49.6	53.0	1.5	0.6	2.4
			CO_2	964.2	27.3	924.4	997.9	0.3	12.2	2.8
Between syringes	A	20	CH_4	45.8	6.4	33.5	53.8	-9.4	1.4	13.9
			CO_2	867.9	126.4	620.2	1017.8	-9.7	28.3	14.6
	B	20	CH_4	49.5	1.8	46.5	52.2	-2.2	0.4	3.5
			CO_2	945.0	29.4	891.0	989.8	-1.7	6.6	3.1
	C	20	CH_4	49.3	1.4	47.4	53.0	-2.5	0.3	2.9
			CO_2	928.1	31.5	884.3	997.9	-3.4	7.0	3.4

S3 Fig provides a graphical representation of the data presented in S2 Table. This clearly highlights the within and between operator and syringe variation associated with closed-loop sample injection for small gas volume testing.

S3 Fig. Comparison of errors for syringe and operator repeatability testing, arranged by syringe and operator (see also S2 Table). Percentage error is $(X_{meas}-X_{exp})/X_{exp}*100\%$. Syringe A presents a worst case, and operator 4 achieved much closer replicates and higher values than the other operators.

Figure S4 compares the noise level on the instrumental signal after mirror cleaning (according to the manufacturers procedure [2] and instrument recalibration (S4 Fig. a and c), and for a worst-case situation (S4 Fig. b and d) following accidental water ingress into the instrument (after drying but not mirror cleaning). In the case presented (S4 Fig. a and c), mirror cleaning achieved RD values close to those expected of a new instrument (RD > 9 µs).

S4 Fig. Extreme ring-down reduction - effect of accidental water ingress on ring down time (RD) for background noise for CH_4 and CO_2 detectors; a. and c. clean mirrors (RD > 9 µs), b. and d. after drying of loop without mirror cleaning (RD < 4 µs).

S5 Fig. Example stacked injection data for sediment incubation headspace gases for paired flasks of sub-samples from increasing depths below surface water-sediment interface.

References

1. Wilkinson J, Maeck A, Alshboul Z, Lorke A (2015) Continuous Seasonal River Ebullition Measurements Linked to Sediment Methane Formation. Environmental Science & Technology 49: 13121–13129.
2. Los Gatos Inc., (2014) Ultra-Portable Gas Analyzer, Mirror Cleaning Procedure, Modell 915-00xx.
